# Stress Responses of *Shewanella*

**DOI:** 10.1155/2011/863623

**Published:** 2011-09-07

**Authors:** Jianhua Yin, Haichun Gao

**Affiliations:** College of Life Sciences and Institute of Microbiology, Zhejiang University, Hangzhou, Zhejiang 310058, China

## Abstract

The shewanellae are ubiquitous in aquatic and sedimentary systems that are chemically stratified on a permanent or seasonal basis. In addition to their ability to utilize a diverse array of terminal electron acceptors, the microorganisms have evolved both common and unique responding mechanisms to cope with various stresses. This paper focuses on the response and adaptive mechanism of the shewanellae, largely based on transcriptional data.

## 1. Introduction

Stress is an inevitable part of the life of all organisms. This is especially true about microorganisms, which reside and thrive in almost all environments on earth, including some considered extremely harsh [1]. Common environmental factors that affect the activities of microorganisms include temperature, pH, water availability, nutrient limitation, presence of various chemicals, osmolarity, pressure, and radiation [2]. Consequently, for every microorganism the ability to adapt rapidly to changes in environments is essential for its survival and prosperity. Regulation that modulates the microbial adaptation to environmental disturbances is rather complex. The most important and efficient control occurs at the level of transcription. Many single stress-induced regulatory circuits have been identified, which enable cells to cope with specific stresses. However, given that microbial cells live in a dynamic environment where multiple factors fluctuate constantly at the same time, stress responses are generally carried out by a regulatory network composed of a series of individual circuits which are highly connected [3]. 

Most of our understanding of microbial stress response mechanisms has come from the study of model microorganisms, particularly *Escherichia coli* and *Bacillus subtilis*. Extensive physiological and genetic analyses of the stress response systems in these two bacteria have helped us to elucidate the complexity of the process, function of critical proteins, and regulation [4]. While model organisms will continue to provide insights into the fundamental properties of the stress response systems, efforts should be extended to other microorganisms, especially those that are of scientific, environmental, and economic importance. 

As one of representatives, the family of *Shewanellaceae* (order Alteromonadales, class *γ*-proteobacteria) is emerging in recent years. The genus *Shewanella* consists of rod-shaped, Gram-negative, aerobic or facultatively anaerobic, polarly flagellated, readily cultivated *γ*-proteobacteria [5–8]. While many *Shewanella* isolates remain uncharacterized, 52 species have been recognized to date [9]. Shewanellae are renowned for its ability to use a diverse range of electron acceptors for anaerobic respiration, including fumarate, nitrate, nitrite, thiosulfate, elemental sulfur, trimethylamine *N*-oxide (TMAO), dimethyl sulfoxide (DMSO), Fe(III), Mn(III) and (IV), Cr(VI), U(VI), As(V), V(V), and others [10, 11]. As a result of this property, Shewanellae have drawn much attention in the fields of bioremediation, biogeochemical circulation of minerals, and bioelectricity [12, 13]. In addition, Shewanellae have now served as the model for ecological and evolutionary studies at the whole genome level because of its diverse habitats and the availability of up to 26 genome sequences [14, 15].

A number of *Shewanella* strains are currently under physiological investigation [11]. However, stress responses have focused nearly exclusively on *Shewanella oneidensis*, the first genome of the shewanellae to be sequenced [16]. The availability of the genome sequence allowed development of high-throughput technologies such as microarrays and proteomics tools, with which an array of assays has been carried out to decipher the ability of *S. oneidensis* to respond to and survive external stresses. While impacts of most of common environmental factors have been examined, oxidative stress imposed by H_2_O_2_ is surprisingly untouched. In this paper, we consider all insights into the stress response mechanisms revealed thus far in *S. oneidensis* and broaden our discussion to other sequenced species if necessary.

## 2. Stress Responses to Temperature Fluctuation

Variation in growth temperature is a common stress encountered in nature. Stress response to sudden fluctuation in growth temperature, has become a model system for studying the impact of environmental stresses on biological systems. The hallmark of this adaptive cellular response is the induction of a limited set of proteins, called Heat shock proteins (Hsps) or Cold shock proteins (Csps). In general, Hsps play important roles in protein folding, degradation, assembly of protein complexes, and transport of proteins across membranes whereas Csps function as RNA chaperons to regulate ribosomal translation, rate of mRNA degradation and termination of transcription [17–19]. 

Using whole-genome DNA microarrays, temporal gene expression profiles of *S.  oneidensis* MR-1 in response to temperature variations have been investigated [20, 21]. Expression profiles indicate that temperature fluctuation has a pleiotropic effect on the bacterial transcriptomes. Both heat and cold shock responses appear to share a couple of common features, including that approximately 15% of the total genes are significantly affected (*P <* 0.05) over a 25-min period, that the global changes in mRNAs are rapid and transient, and that a similar set of proteins are induced to manage energy production and protein damage. For instance, most of genes encoding enzymes in the Entner-Doudoroff pathway and the pentose cycle are highly induced upon a temperature alteration.

**Figure 1:**
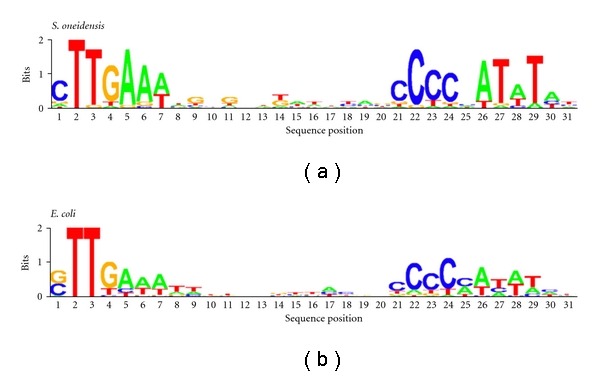
Comparison of consensus *σ* ^32^-recognition sequences of *E. coli* and *S. oneidensis*. The *E. coli* and *S. oneidensis* sequences used were from the published reports by Nonaka et al. [22] and Gao et al. [20], respectively. The sequences were initially aligned by clustalx and the sequence logo was prepared using public software at http://www.bioinf.ebc.ee/EP/EP/SEQLOGO/.

In the case of heat shock response, two lines of evidence suggest that *S. oneidensis *copes with the situation with mechanism similar to that employed by *E. coli*. First, the majority of the genes that showed homology to known Hsps in *E. coli* such as DnaK, DnaJ, GroEL, GroES, GrpE, HtpG, and Lon/La proteases were highly induced. Second, the identified *σ* ^32^ consensus sequences (CTTGAAA-13/15bp-CCCCAT) of both bacteria for heat shock gene promoters are virtually the same (Figure 1), indicating that the induction of most Hsps owns to a rapid and transient increase in the intracellular concentration of an alternative *σ* factor, *σ* ^32^ encoded by *rpoH*. Nevertheless, novel findings are not scarce. After numerous attempts, we failed to remove *rpoH* from the genome, implicating that *σ* ^32^ is essential in *S. oneidensis* (unpublished result). Additionally, some hypothetical proteins (i.e., SO2017) are under the control of *σ* ^32^, suggesting that *S. oneidensis* recruits new proteins to overcome increased temperature (Table 1).

Unlike *E.  coli*, most *Shewanella* strains are psychrotolerant. In terms of the canonical Csps* S. oneidensis* possesses three (of which two (SO1648 and SO2787) are cold inducible) whereas *E. coli* has nine (of which four are cold inducible) [19]. Both SO1648 and SO2787 are important in growth at low temperatures evidenced in the mutational analysis [21]. The *S. oneidensis *genome carries two more genes encoding Csd(cold shock domain)-containing proteins (SO0733, 203 aa; SO1732, 224 aa) whose C-terminal lacks sequence similarity to any known proteins. Intriguingly, such a structure has been found only in eukaryotes, with the exception of *Mycobacterium* [24]. Neither SO0733 nor SO1732 is found to be induced upon a decrease in temperature or influences growth at low temperature, indicating that these Csd-containing proteins may not be involved in cold stress response. 

*S.  piezotolerans *WP3 is another *Shewanella* that has been studied in respect of response to low temperatures. Strikingly, none of its Csps are cold inducible, suggesting that these proteins may not play an indispensible role in the process [25]. Instead, the organism utilizes other strategies to overcome temperature downshifts. These include increased production of EPA (eicosapentaenoic acid) and BCFA (branched-chain fatty acid) [26], induced expression of RNA helicase DeaD which may facilitate transcription, morphological changes in cell membrane, and elevated assembly of lateral flagella (The organism possesses both polar and lateral flagella.) [25]. In addition, a novel filamentous phage (SW1) is found to be significantly induced at low temperature but the significance of this event in the cold adaptation of *S. piezotolerans *WP3 is unknown [27].

## 3. Stress Responses to Acidic and Alkaline pH

Microorganisms live in a volatile environment where extracellular pH changes frequently. To minimize the acid- or alkaline-induced damage, various adaptive strategies have evolved [28, 29]. Studies on *E.  coli* have revealed that bacterial cells activate outward H^+^ pumps such as K^+^/proton antiporters in response to acute cytoplasmic acidification and sodium proton antiporters, which bring in 2 H^+^ for each Na^+^ extruded, to adapt to alkaline pH in the presence of Na^+^. To survive upon prolonged acid stress exposure, cells rely on the arginine and glutamate decarboxylase/antiporter systems, which are thought to counteract external acidification through the consumption of intracellular protons and the generation of alkaline amines. Additional acid tolerance responses include regulation of proton permeability by induction of membrane proteins and lipid modification enzyme. In the case of alkaline stress, amino acid metabolic enzymes such as tryptophan deaminase (TnaA) and *o*-acetylserine sulfhydrylase A (CysK) are induced to reverse alkalinization by metabolizing amino acids to produce acidic products.

The response of *S. oneidensis* to acid and alkaline stresses intersects with other stresses evidenced by elevated expression of RpoS, a central regulator of stationary-phase gene expression [30]. It is reasonable to speculate that *S. oneidensis* cells upon altered pH mimic those at the stationary phase. In respect of response to acidic pH, the mechanism of *S. oneidensis* is fundamentally different from that of *E. coli*. The most important and effective player of *E. coli* in mediating acid resistance is the glutamate-dependent (Gad) system, which is missing in all sequenced *Shewanellae* [31]. Additionally, none of genes encoding H^+^ ex-pumps are found to be induced. Instead, proteins showing substantial induction are rather diverse, including those functioning in cell envelope structure (e.g., *csg* genes), glycogen biosynthesis (*glg* operon), fatty acid metabolism (*fadBA*), glutamate synthesis (*gltBD*), phosphate transport (*so1724* and *pstB-1*), and regulation (e.g., *rpoS* and *phoU*). This observation indicates that the molecular effects of acute acidic pH are profound and multifarious. Upon alkaline pH, as in *E. coli* Na^+^/H^+^ antiporter systems (NhaA) are particularly important in maintaining a pH-homeostatic mechanism, thus enabling *S. oneidensis* to survive and adapt to external alkaline conditions.

## 4. Stress Responses to Osmolarity

The bacterial response to hypertonic stress includes a range of mechanisms. The most important one is regulation of aquaporins in the outer membrane for water intake by the stationary-phase sigma factor, RpoS [32]. It is common that upon the stress condition K^+^ uptake is activated and K^+^ ions are maintained at high levels. Additionally, cells accumulate neutral, polar, small molecules, such as glycine betaine (GB), proline, trehalose, or ectoine [33]. These compatible solutes serve as osmoprotectants and are synthesized and/or imported into the cell. Many *Shewanella* species are marine microorganisms and therefore are naturally tolerant to relatively high levels of salt. Although some like *S. oneidensis*, are obtained from freshwater environments, they are able to grow in the presence of up to 0.6 M NaCl [34]. 

The primary response of *S. oneidensis* to hyperosmotic conditions is similar to *E. coli*. Genes encoding K^+^ uptake proteins, Na^+^ efflux system components, and glutamate synthesis are found to be highly induced. Nonetheless, some novel mechanisms are observed. Genes encoding proteins involved in accumulation of compatible osmolytes are either missing in the genome or transcriptionally unaffected when encountered stress. Interestingly, genes encoding TCA cycle are particularly active, probably producing much needed ATP for ion transport. This may also explain that *S. oneidensis* shows reduced motility and chemotaxis responding capability under the stress given that the assembly of flagella is extremely energy consuming [34].

## 5. Stress Responses to Radiation

Radiation is potentially lethal and mutagenic to all organisms. Although DNA is the major chromophore in general, effects of radiation are in fact pleiotropic [35, 36]. *S. oneidensis*, one of the most radiation-sensitive organisms known so far, is approximately 1 order of magnitude more susceptible to all wavelengths of solar UV, UV, and ionizing radiation than *E. coli* [35, 37–40]. This is strikingly because the organism similar to *E. coli* possesses the complete set of genes for photo-reactivation, and nucleotide excision repair, and SOS response, primary mechanisms that protect cells from DNA damages and radiation-induced oxidative stress [16, 41, 42]. All of these *S. oneidensis *genes appear to be functional and crucial in the cellular response to radiation, supported by significant upregulation in transcriptional analyses. It is interesting to note that *Shewanella* strains vary significantly in their susceptibility to radiation although compared to *E. coli* they are still much less resistant. The general trend is that the more radiation exposure is in the habitat where the organisms are isolated the less sensitive they are [37]. For instance, *S. oneidensis* MR-1 from lake sediment and *S. putrefaciens *200 from a crude oil pipeline are more sensitive to radiation than *S. algae* from the surface of a red alga and *S. oneidensis* MR-4 from the surface of the Black Sea [33].

It has been suggested that the hypersensitivity to radiation may be in part due to the activation of prophage [38–40]. Radiation has been used as a standard approach to induce prophage in a variety of bacteria [43, 44]. In *S. oneidensis*, upon radiation the majority of LambdaSo, MuSo1, and MuSo2 genes are induced and phage particles have been found in the cultures, indicating that a great number of cells are lysed by lytic phages. It has also been implicated that a large number of iron-containing proteins may be partially accountable for the susceptibility. Compared to *E. coli* which hosts only five to seven cytochrome *c* proteins, *S. oneidensis* contains 41 such proteins, some of which are electron transport proteins and essential in respiration [45, 46]. Damages on these proteins by reactive oxygen species (ROS) generated in cells upon radiation would likely cause two detrimental results [47]. First, damaged proteins *per se* may be dysfunctional, directly reducing ability to survive or thrive. Second, damaged proteins release irons into cultures, which further induce ROS production [48]. This second wave of ROS may be more fatal because it comes at the onset of recovery of seriously damaged cells. Furthermore, the finding that the intracellular Mn/Fe concentration ratios correlate well with resistance to radiation may explain the hypersensitivity of *S. oneidensis*, which has the lowest ratio among bacteria tested so far [35, 49].

## 6. Stress Responses to Heave Metals

Many of metal elements are required for microbial growth mostly as cofactors in metabolic pathways. However, they exert deleterious effects under conditions of elevated concentration [50]. *Shewanellae* have attracted much attention because of their ability to reduce metal ions including chromium, cobalt, iron, manganese, technetium, uranium, and vanadium, some of which are not needed and highly toxic for most organisms [10, 51, 52]. At the low level these metal ions are taken as electron acceptors by cells and mildly induced some stress-associated genes [53]. However, at the high concentration some of them elicited a distinctively different pattern [54–60]. The cellular resistance mechanisms displayed by microorganisms are diverse and include biosorption, diminished intracellular accumulation through either direct obstruction of the ion uptake system or active chromate efflux, precipitation, and reduction of metals to less toxic form. Multiple regulatory circuits are found to work together to cope with the stress response of *S. oneidensis* to heavy metal compounds. The major ones include those modulating oxidative stress protection, detoxification, protein stress protection, iron acquisition, and DNA repair [50].

The molecular response of *S. oneidensis* to heavy metal shock elicits a distinctively different transcriptional profile compared with metal reduction [53–60]. This observation is consistent with that metal reduction and toxicity resistance mechanisms are to be unlinked cellular processes [61]. Responses of *S. oneidensis* to acute stresses imposed by a variety of heavy metals share a common strategy: survive first and then exert both general and specific stress responses. As a result, *S. oneidensis *up-regulates its resistance-nodulation-cell division (RND) protein family genes that facilitate cation export and thus confer heavy metal resistance. Once the first line of defense is initiated, cells employ both general and specific stress responses that are inseparable from each other to recover from the crisis. Alternative sigma factors including RpoS, RpoH, RpoE, along with stress-response-related genes are induced, leading to induction of a variety of detoxification, resistance, and transport functions. Such coordinated expression of stress response and detoxification mechanisms in *S. oneidensis* may offer an advantage to thrive in anoxic metal-reducing conditions in aquatic sediment and submerged soil systems where substantial amounts of heavy metals can be generated. 

Two specific responding mechanisms are particularly worth noting. The first is that genes/proteins involved in iron transport are transcriptionally active and implicated to play an important role in the process. Although induction of siderophore biosynthetic and iron transport genes may not be a direct consequence of intracellular iron limitation, several lines of evidence suggest that it is more likely to be indirect by interfering with the Fur (ferric uptake regulator) protein, which eventually results in derepression of the iron regulon. Several reports have demonstrated that Co^2+^, Mn^2+^, or other divalent cations interact with the Fur-binding sites [62, 63]. Moreover, iron-chelating siderophores from other microorganisms have been shown to be able to bind other metals, such as thorium, uranium, vanadium, and plutonium [64, 65]. By increasing siderophore production, cells can reduce toxicity of heavy metals by sequestration. The other is that sulfur transport and assimilation is promoted. While the underlying mechanism is currently unknown, an explanation is offered. In *S. oneidensis*, reactive oxygen species (ROS) produced in cells by heavy metal stresses can damage iron-containing proteins. As cysteine residues in these proteins are essential to their functions, an extra mount of cysteine is needed for protection. To this end, cells elevate transportation of inorganic sulfate which is reduced and incorporated into bioorganic compounds via assimilatory sulfate reduction, which is the major route of cysteine biosynthesis in most microorganisms [66].

## 7. Concluding Remarks

As a potential strategy for the reductive immobilization or detoxification of environmental contaminants, *in situ* bioremediation has received much interest and attention in last 20 years and are becoming more prevalent today. As its intrinsic feature, the application puts its work force, mostly bacteria, “*in situ*” facing the unpredictability of individual microbial processes and constant fluctuations in environments. Thanks to the availability of the *S. oneidensis* genome sequence, stress responses of the microorganism have been extensively investigated, generating a handful of insights into mechanisms adopted to cope with detrimental conditions. Nonetheless, adaptive mechanisms of *Shewanella *to environmental stresses are still a large playing field for three reasons. First, a number of common stressful agents, especially reactive oxygen species, are not visited. Second, the complex components and regulation in the bacterial stress responses discussed in this paper are mostly based on transcriptional profiling and thus experimental validation is urgently warranted. Last, but definitely not the least, the genus is composed of members which are not only isolated from extremely diverse habitats but also lack unifying phenotypic features, prompting exploration to be extended to other ecological groups of the Shewanellae.

## Figures and Tables

**Table 1:** Genes predicted to be under the direct control of *σ* ^32^ in *S. oneidensis. *

Locus	Gene	Product	Start	End	Sequence	Weight
SO2016	*htpG *	heat shock protein HtpG	−84	−55	CTTGAAAAGTGGATTTGCAGCCCCATTTTA	20.3
SO4162	*hslV *	ATP-dependent protease HslV	−83	−54	CTTGAATTCTGGCTATCCATCCCCATATTT	20.1
SO1126	*dnaK *	chaperone protein DnaK	−78	−48	CTTGAAAAAAAATGCGTCCGGCCCCATATCT	18.6
SO0406	*trxA *	thioredoxin 1	−80	−51	CTTGAAAAGCTATTTTTCAGCCCCAATATA	18.4
SO1524	*grpE *	heat shock protein GrpE	−74	−45	CTTGAAACGTCAAAATTGATCCCCATAATA	18.2
SO2593		conserved hypothetical protein	−262	−232	CTTGAAATGGGGAGTTTAACTCCCCATTTTT	17.9
SO3577	*clpB *	clpB protein	−77	−48	CTTGAATTTGGTTAAATAGCCCCCATCTTT	16.8
SO0452	*trxC *	thioredoxin 2	−60	−31	CTTTAAATTCGCCGCAGCGCCCCCATATCT	15.7
SO2017		conserved hypothetical protein	−106	−76	CTTGAGTTGAGACGCAAGTGCCCCGATTTAC	14.4
SO1796	*lon *	ATP-dependent protease La	−68	−39	ATTGAAAGGGCATAAACCGCCCCAATATAC	14
SO2277	*ibpA *	16 kDa heat shock protein A	−167	−138	CTTGAAATCCGTTTTCCTATCCTTATATCT	13.5
SO0703	*groES *	chaperonin GroES	−123	−93	CTTGGATCTGGCGGGGGTGAACCCCATATCA	13.3
SO4492		conserved hypothetical protein	−76	−48	GTTGAAAAGAATTGATTTGCCCCAAGATA	12.8
SO1794	*clpP *	ATP-dependent Clp protease, proteolytic subunit	−83	−55	CTTGACTTGATTAGCAGTTCGCCATTTAT	12.8
SO1163		conserved hypothetical protein	−60	−31	CTTGAATCGGGTATAATCGCCACCATATAG	12.7
SO3863	*modA *	molybdenum ABC transporter, periplasmic molybdenum-binding protein	−206	−177	CTTGAGTAAATGTTATTGTCCCCGATCAAT	12.3
SO1196	*rrmJ *	ribosomal RNA large subunit methyltransferase J	−65	−36	GTTGAAAAACCGCTATTCTACCCTTATATA	12.2
SO2723		HIT family protein	−47	−17	ATTGAATTGCTAGTATACTATCCCAATTAAC	11.8
SO1213		hydrolase, TatD family	−240	−211	GTTTAAAGGCGGTGATTCACCGCCTTTTTT	11.8
SO2705	*topA *	DNA topoisomerase I	−77	−49	CTTGAAACTCTCAGTGCAACCCTCTATAT	11.1
SO3501		conserved hypothetical protein	−297	−268	CATGAATTTGGCAACGGCACCGCCATTTTC	11
SO2728	*htpX *	peptidase HtpX	−101	−71	GTAGAAAAACTCTTATCTTTACCCCTTGAAT	10.6
SO1473	*smpB *	SsrA-binding protein	−69	−39	GTTGAAATAGCTCAAATAAACCCTTATATCC	10.3
SO0698	*fsxA *	fxsA protein	−64	−34	CTTGAATTAAGACCGGATTGCCCCCATTTAG	10.3
SO3402		hypothetical protein	−396	−367	ATTGAAAAGGGCCTTTATGGCCCTTTTTCG	10.2
SO1937	*fur *	ferric uptake regulation protein	−164	−135	CTTGAATTGCCGCAATTTATTGCAATTTCA	10.2
SO2706	*astB *	succinylarginine dihydrolase	−40	−11	TTTGAATAAATAATAACCTTCCCTATCACA	9.7
SO0868		hypothetical protein	−93	−63	GTTTAAATGGGGAGAAAACAACTCCATTTTA	9.4
SO3961	*rpoN *	RNA polymerase sigma-54 factor	−83	−53	CTTGAATTTGGCAGCGCAAAGCGCCATCAGT	9.4
SO0930	*tkt *	Transketolase	−161	−133	CTTGAATAGTTCATCCTTAAGCCATTTTT	9.3
SO3528		hypothetical protein	−195	−167	AATGAAAAGAGGCTTTTAGCCTCTTTTTT	9.3
SO1580		TonB-dependent heme receptor	−57	−28	CTTTGATGCCTATAATGCCGCCCTATTTTT	9.3
SO2314		ISSo1, transposase OrfA	−227	−197	GTTAAAATGACAAGCATGGAGCGCAATATCT	9.2
SO1903		hypothetical protein	−71	−42	TTTGGGATTATTTAATTCCCCCCCATTTAT	9.2
SO1097		conserved hypothetical protein	−63	−33	CATGAAATCTGCGATAATCAGCGCCTTATTT	9.2
SO0595		hypothetical protein	−327	−298	CTTGATTAGAGCCACGTCGCTCCAATTTTT	9.2
SO4719		conserved hypothetical protein	−44	−16	CTAGGCATTTGAGTTGGAACCCTATTTTT	9.1
SO4287	*motA *	chemotaxis motA protein	−127	−99	CTTGAATTTAGTAGATTTTCCTTATAATG	9.1
SO3113	*tgt *	queuine tRNA-ribosyltransferase	−96	−67	GTTGAACCTTTTAGATCTGTCCCTATCTCT	9

Genome screening with *σ* ^32^ weight matrix is performed using RSAT at http://rsat.ulb.ac.be/rsat/RSAT_home.cgi [23]. Genes with a weight score over 9 are shown.
